# Defining Optimal Aerobic Exercise Parameters to Affect Complex Motor and Cognitive Outcomes after Stroke: A Systematic Review and Synthesis

**DOI:** 10.1155/2016/2961573

**Published:** 2016-01-10

**Authors:** S. M. Mahmudul Hasan, Samantha N. Rancourt, Mark W. Austin, Michelle Ploughman

**Affiliations:** Recovery & Performance Laboratory, Faculty of Medicine, Memorial University, L.A. Miller Centre, Room 400, 100 Forest Road, St. John's, NL, Canada A1A 1E5

## Abstract

Although poststroke aerobic exercise (AE) increases markers of neuroplasticity and protects perilesional tissue, the degree to which it enhances complex motor or cognitive outcomes is unknown. Previous research suggests that timing and dosage of exercise may be important. We synthesized data from clinical and animal studies in order to determine optimal AE training parameters and recovery outcomes for future research. Using predefined criteria, we included clinical trials of stroke of any type or duration and animal studies employing any established models of stroke. Of the 5,259 titles returned, 52 articles met our criteria, measuring the effects of AE on balance, lower extremity coordination, upper limb motor skills, learning, processing speed, memory, and executive function. We found that early-initiated low-to-moderate intensity AE improved locomotor coordination in rodents. In clinical trials, AE improved balance and lower limb coordination irrespective of intervention modality or parameter. In contrast, fine upper limb recovery was relatively resistant to AE. In terms of cognitive outcomes, poststroke AE in animals improved memory and learning, except when training was too intense. However, in clinical trials, combined training protocols more consistently improved cognition. We noted a paucity of studies examining the benefits of AE on recovery beyond cessation of the intervention.

## 1. Introduction

Most people admitted to hospital with stroke continue to have enduring motor and cognitive deficits that interfere with their previous roles and quality of life [1]. Very few people admitted with stroke regain functional use of the hemiplegic arm and hand [2] and recent research suggests that there is a limited time window in which to make the greatest gains [3]. During this window of neuroplasticity, the patient must practice therapist-guided task-specific movements, to drive Hebbian plasticity in order to regain function [4]. However, after stroke, cognitive health and the recovery (or relearning) of complex motor skills are intertwined. Relearning of complex movement, which is fundamental to neurorehabilitation, requires most cognitive domains including working memory [5], attention [6], and executive function [7]. Motor learning is impeded by cognitive impairment [8], limiting the ability of the stroke patient to understand, repeat, refine, and analyze recovering movement [9].

Aerobic exercise (AE) is one intervention recommended as part of stroke best practices to improve gait and cardiovascular fitness [10, 11]. Researchers are beginning to explore how AE, by increasing neurotrophins and blood supply to the brain, could also improve other outcomes (unrelated to fitness and gait) such as cognition and relearning of complex skills [12]. Two recent meta-analyses confirmed that AE enhanced cognitive performance, at least in healthy populations [13, 14]. However, based on the findings of two systematic reviews [8, 15], there was limited evidence to support the use of AE to improve cognition in neurorehabilitation practice. More compelling findings have been reported in animal models of stroke, suggesting that rigorous preclinical and clinical trials are still warranted [16].

AE is defined as “planned, structured repetitive physical activity for extended periods and at sufficient intensity to improve or maintain physical fitness” [11]. To improve physical fitness it is recommended that AE be instituted most days of the week at gradually increasing intensity for at least 8 weeks [11]. Whether the AE parameters to improve physical fitness are the same as those to improve cognition and relearning of complex skills after stroke is not known. In two previous reviews examining mechanistic effects of AE on the brain, we reported that, in animal models, moderately intense forced AE, instituted soon after stroke (24–48 hours), enhances neurotrophins, synaptogenesis, and dendritic branching and protects perilesional tissue against oxidative damage [69, 70]. Whether these training parameters translate into improved cognition and relearning of complex motor skills (in animal models or clinical trials) is not known, important information in order to design future clinical and preclinical studies. Researchers would be concerned about AE parameters: frequency, intensity, duration, mode (i.e., treadmill, swimming, or bicycling), and the timing of exercise onset after stroke. In addition to optimizing parameters, scientists must also target outcomes responsive to AE since AE may not affect all cognitive and motor domains in the same way, if at all.

We undertook this review to consolidate potentially important findings in both animal models and clinical trials testing the effects of AE on cognitive and complex motor performance after stroke. We hoped to gain methodological insights to inform future preclinical and clinical studies investigating this potentially promising area of neurorehabilitation.

## 2. Methods

### 2.1. Search Methods

This review is a component of a larger systematic review of human and animal studies on the effects of AE on repair, recovery, and plasticity after stroke [69, 70]. We searched PubMed, CINAHL, PsycINFO, the Cochrane Library, and the Central Register of Controlled Clinical Trials using controlled vocabulary (as shown below) supplemented with keyword searching to ensure that the search was exhaustive. Searches were limited to English language articles but were not limited by study type (i.e., randomized controlled trials) in order to retain animal studies. We retrieved new publications until July 16, 2014. Once titles and abstracts of studies retrieved in the search were merged into a single database and duplicates removed, all four review authors, in teams of two, independently evaluated the titles, abstracts, and full texts against inclusion criteria. Discrepancies in data collection were resolved through consensus. In instances where full-text manuscripts were unavailable, corresponding authors were contacted and requests for full texts made. We hand-searched reference lists of studies that met inclusion criteria for additional potentially relevant studies. Data was extracted and recorded on standardized data collection forms and results were categorized by outcome.

Our goal was to synthesize and identify commonalities among all the included studies: those in animal models, case studies, preliminary clinical studies, and randomized controlled trials. Rather than assessing study quality or risk of bias, we identified trends when two or more studies demonstrated a similar result.

*Search Terms for PubMed, CINAHL, PsycINFO, the Cochrane Library, and the Central Register of Controlled Clinical Trials*

*#1: “Cerebral Hemorrhage”[MeSH] OR “Intracranial Hemorrhages”[MeSH] OR “Stroke”[MeSH] OR “Brain Ischemia”[MeSH] OR “Brain Hemorrhage, Traumatic”[MeSH] OR “Cerebral Hemorrhage, Traumatic”[MeSH] OR “Brain Infarcion”[MeSH] OR “Cerebral Infarction”[MeSH] OR “Infarction, Middle Cerebral Artery”[MeSH]*

*#2: “cerebral hemorrhage” OR “brain hemorrhage” OR “cerebral ischemia” OR “brain ischemia” OR “cerebral infarction” OR “brain infarction” OR stroke OR “cerebrovascular accident”*

*#3: #1 OR #2*

*#4: “Exercise”[MeSH] OR “Motor Activity”[MeSH] OR “Exercise Therapy”[MeSH] OR “Physical Fitness”[MeSH] OR “Motor Skills”[MeSH] OR “Physical Conditioning, Animal”[MeSH]*

*#5: exercise OR aerobic OR “physical activity” OR treadmill OR running OR “therapeutic exercise” OR “physical fitness” OR “physical conditioning” *

*#6: #4 OR #5*

*#7: “Executive Function”[MeSH] OR “Cognition”[MeSH] OR “Neuronal Plasticity”[MeSH] OR “Cognition Disorders”[MeSH] OR “Mental Processes”[MeSH] OR “Recovery of Function”[MeSH] OR “Memory”[MeSH] AND “Brain-Derived Neurotrophic Factor”[MeSH]*

*#8: “executive function” OR “cognitive function” OR cognition OR cognitive OR neurocognitive OR neuroplasticity OR “neuronal plasticity” OR “mental processes” OR memory OR “recovery of function” OR “motor performance” OR neurotrophic*

*#9: #7 OR #8*

*#10: #3 AND #6 AND #9*

*#11: Search (#10) Filters; English*

*#12: #3 AND #6 AND (“rats”[MeSH Terms] OR “rats”[All Fields] OR “mice”[MeSH Terms] OR “mice”[All Fields] OR “animals”[MeSH Terms:noexp] OR animal[All Fields] OR “animals”[MeSH Terms:noexp] OR animals[All Fields]) Filters: English*

*#13: #11 OR #12*

### 2.2. Inclusion/Exclusion Criteria

#### 2.2.1. Subjects

Clinical studies were limited to those investigating adults with confirmed ischemic or hemorrhagic stroke of any duration (acute or chronic). Animal studies were included if they employed a model of stroke: incomplete global ischemia (i.e., two-vessel occlusion), focal cerebral ischemia (endothelin-1-induced occlusion, middle cerebral artery occlusion, and photothrombosis), and intracerebral hemorrhage (i.e., collagenase injection-induced hemorrhage).

#### 2.2.2. Intervention

AE intervention included any type of exercise of any intensity, duration, or frequency that was aimed at improving cardiorespiratory fitness. In humans, the study must have included some measure of intensity, such as heart rate or perceived level of exertion, and the intervention must have extended beyond two minutes in order to pass the anaerobic metabolism threshold [71]. In animal models, AE included both voluntary exercise (VE, i.e., placement in cage with a VE wheel) and forced exercise (FE, i.e., placement on a moving treadmill away from an electrified grid, motorized running wheel, swimming, and placement on a rotarod) for at least 20 minutes. We divided animal FE paradigms into low intensity (<10 m/min), moderate intensity (10–17 m/min), and vigorous intensity (>17 m/min) based on previous research [70]. We reported the intensity of exercise in clinical trials as described by the authors of the included studies.

#### 2.2.3. Outcomes

We included human and animal studies that measured at least one domain of cognition or complex motor performance after an exercise intervention that included AE. Since AE is often delivered using locomotion and task-specific training usually improves the target task [72], we excluded locomotor outcomes such as gait velocity. We were interested in cognition and more complex movement such as lower limb coordination, balance, and skilled reaching.

## 3. Results 

**Figure 1:**
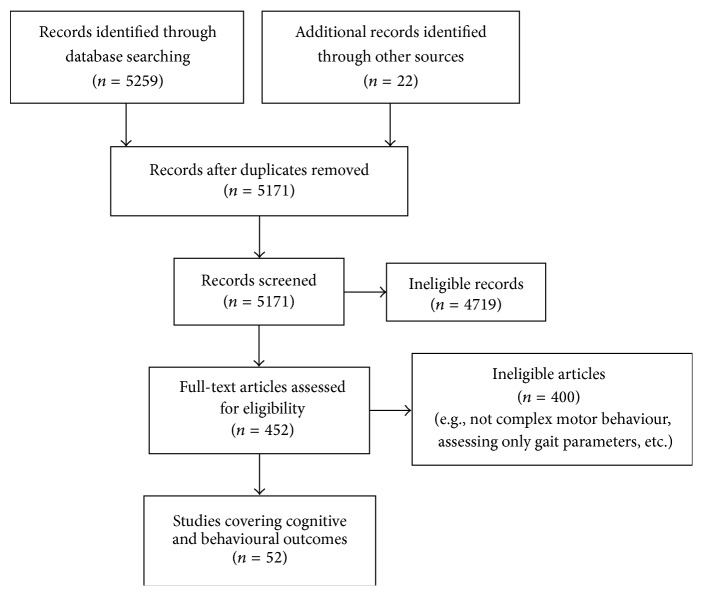
Flow diagram of the study selection process.

A total of 5,259 titles were returned from the literature search. Following review of titles, abstracts, and manuscripts and lastly a hand search of reference lists, 52 articles were included in this review (Figure 1). We grouped and summarized the findings according to the specific outcome tested (Table 1) in animal models (*n* = 35) and in human trials (*n* = 17).

Studies included in this review utilized multiple outcome measures: balance and lower extremity coordination (e.g., climbing, beam walking, and rotarod testing), upper limb movement (e.g., gross upper limb performance, skilled limb movements), and cognitive performance (memory, learning, and executive control, Table 1). The optimal training parameters to affect these outcomes, distilled from the included animal and clinical studies, suggest that the optimal parameters to improve cognition and complex motor outcomes depend on the specific outcome measured (Table 2). For example, in terms of balance and lower extremity outcomes, consistent findings in animal and clinical studies suggest that any mode of AE, repeated frequently (3 or more times per week) at moderate-to-vigorous intensity, instituted during the subacute phase after stroke, can improve these outcomes. Conversely, we found that there was insufficient consistency in results to suggest that AE would influence upper limb control. Synthesized findings are described in Table 2.

### 3.1. Motor Performance

#### 3.1.1. Balance and Lower Limb Coordination

**Figure 2:**
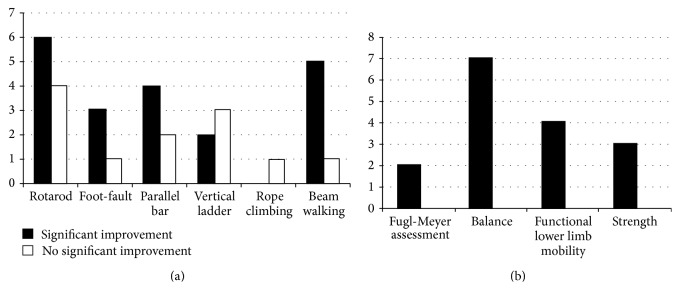
Effects of AE on balance and locomotor/lower limb coordination. (a) Animal studies (*n* = 22) are presented as the frequency of AE-induced positive (black bars) and negative (white bars) findings. (b) Clinical trials (*n* = 9) predominantly reported positive findings (black bars) in both experimental and control groups: outcomes included Fugl-Meyer assessment, balance (e.g., Berg Balance Scale, limits-of-stability test), functional lower limb mobility (e.g., get-up and go test, sit-to-stand test, and turning speed), and strength.

Thirty-one studies, twenty-two of which involved animal models of stroke, examined the effects of AE on balance and locomotor/limb coordination (Figure 2(a)). Synthesized data from animal studies suggests that the timing of exercise initiation after stroke affects motor outcomes (Table 2). Eleven studies of early-initiated FE (within 5 days) of low-to-moderate intensity (8–20 m/min, 20–30 min, 5–7 days per week) reported improved limb coordination during balance and complex locomotor tasks [17–19, 21–24, 31, 32, 37, 38]. Less consistent results were found when assessing late onset FE (5–7 days after stroke) in animal models. Both low intensity (8 m/min) and progressing intensity (2–20 m/min) FE beginning 5–7 days after stroke failed to improve lower limb function [20, 28]. Only one study compared early- and late-initiated exercise after stroke and they [33] found that both early (24 hours) and late (7 days) training groups regained prestroke motor function by experiment termination, albeit the late onset FE group initially exhibited decreased balance performance.

In addition to the timing of the initiation of exercise, the duration of the intervention also affected functional motor outcome (Table 2). FE-based interventions that either were greater than four weeks in duration [29] or were less than four days in duration [30] did not result in significant improvements in balance and lower limb function in animals (Figure 2(a)).

In terms of VE, 5–7 days of wheel-running starting 2 days after stroke significantly improved balance and lower limb coordination [34–36]. However, VE that did not include prestroke exercise habituation [25, 26] or involved much longer protocols (13 weeks) [27] did not demonstrate significant improvements in limb coordination when compared to controls. This may suggest that the animals may have benefited from orientation to the training before stroke to reduce stress and that long duration VE at the same level of intensity may not offer animals adequate challenge or motivation.

We included nine studies that examined the effects of AE on balance and complex lower extremity outcomes in people about four or more months after stroke (Figure 2(b), Table 2). We excluded studies that assessed only gait parameters, based on our selection criteria (Figure 1). Four studies reported significantly improved lower limb recovery and balance following four weeks of 20–30-minute cycling or body weight supported treadmill training [42, 43, 45, 46]. Likewise, eight weeks of moderate AE improved functional lower limb mobility after training while balance was significantly improved at follow-up 8 weeks later [41]. Longer duration programs (3 months) consisting of thrice weekly physiotherapist-supervised brisk walking [44] also significantly improved balance. Synthesized data from these studies suggest that although a variety of balance and complex lower extremity motor outcomes were assessed (Figure 2(b)), AE always resulted in improvements, regardless of the type, intensity (>mild), and duration (>4 weeks) of the interventions.

Interestingly, only three clinical trials compared AE to a control intervention [40, 41, 47]. Jin and colleagues [40] reported that while 12 weeks of moderate intensity aerobic cycling improved balance and lower extremity strength, the control group, which received conventional therapy, also exhibited improvements at the end of the trial. Furthermore, both a home-based exercise program and AE resulted in greater balance and lower limb recovery compared to usual care with no differences between the intervention groups [47]. Therefore, while most studies employing a pre/posttest design reported positive balance and lower extremity outcomes, studies comparing AE to a control intervention suggest similar improvements in both groups.

One issue in stroke rehabilitation is the concept that there is an optimal therapeutic window for recovery to take place [73]. We found two studies which compared the effects of late- versus early-initiated AE. Straube and colleagues [39] implemented a 10-week high intensity progressive program, consisting of treadmill training, overground walking, and stair climbing among acute (1–6 months) and chronic (>6 months) stroke participants. Both the early and late groups exhibited significantly improved balance, with no differences between the groups. Similarly, four weeks of body weight supported treadmill training followed by general exercise improved lower limb motor performance among people with recent (<6 months) and chronic (>12 months) stroke [46]. These two studies suggest that AE interventions improved lower limb motor outcomes and balance in clinical populations, regardless of the timing of the intervention after stroke; however neither implemented a control group.

#### 3.1.2. Upper Limb Movement

**Figure 3:**
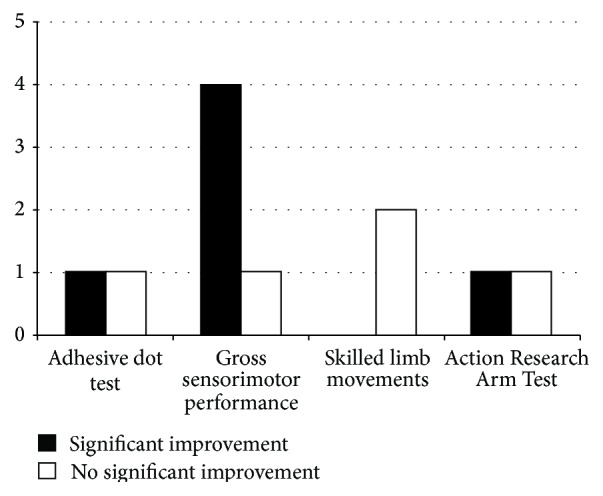
Effects of AE on upper limb movement: significant improvements (black bars) and negative findings (white bars) in animal studies (*n* = 7) and clinical trials (*n* = 2) were subcategorized under specific tasks: adhesive dot test, gross sensorimotor performance (e.g., forelimb placement test, Garcia index, or the cylinder test), and skilled limb movements (e.g., staircase test). The clinical trials included in this review only assessed performance on the Action Research Arm Test as a measurement of skilled movement of the upper limb.

Nine studies investigated the effects of AE on gross sensorimotor or skilled upper limb function on the stroke-affected side (Figure 3, Table 2): seven involving animals [20, 29, 48–52] and two involving humans [53, 54].

In animal models of focal ischemia, effects of AE on upper limb movements were promising, although the outcomes measured were diverse. Three weeks of high intensity FE initiated 24 h after stroke significantly improved performance on a forelimb placement test [50, 51] or the Garcia index [49], both being measures of gross sensorimotor upper limb function. Ten days of VE also resulted in improved performance on the adhesive dot removal test (sensory) as well as the cylinder test (gross motor) [52]. However, two weeks of mild progressing to moderate intensity FE, beginning either 24 h or one week after intracerebral hemorrhage, did not significantly improve performance on the adhesive dot removal test [20].

In addition to gross upper limb function, the effects of AE on performance of skilled upper limb tasks have been assessed in two animal studies and two clinical trials (Figure 3). Six weeks of VE [48] or five weeks of moderate AE [29] did not improve performance of skilled reaching and grasping. However, Ploughman and colleagues [29] demonstrated that skilled reaching training or the pairing of AE with reaching, rather than running alone, improved performance of a forelimb task. Interestingly, the combined effects of AE and reaching surpassed that of reaching alone, suggesting a synergistic positive effect.

The two clinical trials assessing complex upper limb motor skills showed conflicting results. Among people with acute stroke (<4 weeks), twelve sessions of arm ergometry (0 resistance, 55–60 rpm, for forty minutes) did not significantly improve performance of the Action Research Arm Test, an 18-item test of arm and hand tasks, using the affected hand [54]. However, in humans with chronic stroke, a single bout of BWSTT (20 minutes of “somewhat hard” intensity, including a 5-minute warm-up and a 5-minute cooldown) resulted in significant improvements in the same test [53]. The authors proposed that the apparent improvement may have been due to short term relaxation of spasticity caused by the rhythmic motion of the treadmill.

In summary, AE seems to positively influence gross sensorimotor recovery but not upper extremity or forelimb skilled task performance after stroke (Figure 3, Table 2). However, when AE and skilled training are combined, the results are more promising. It is important to consider that, in animals, the forelimbs are employed during the AE intervention, which could account for the recovery observed. In contrast to animal models, clinical trials are inconclusive and there is a need for developing long duration (>4 weeks) protocols with follow-up to demonstrate the durability of benefits.

### 3.2. Cognitive Performance

**Figure 4:**
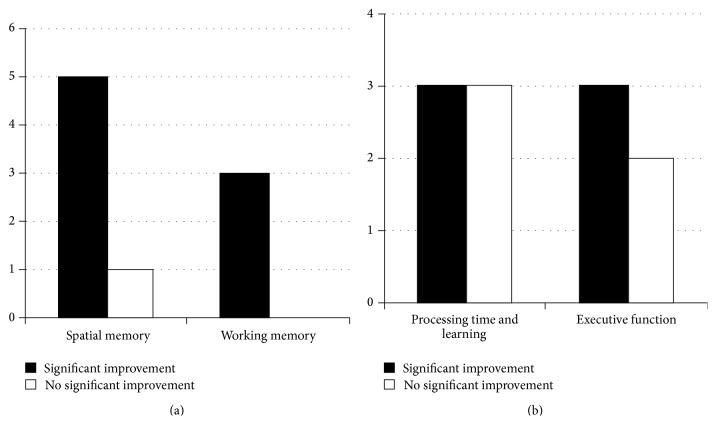
Effects of AE on cognitive performance: frequency of AE-induced positive (black bars) and negative (white bars) findings in animal studies (a) investigating spatial memory (*n* = 6) and working memory (*n* = 3) and clinical trials (b) assessing processing time and learning (*n* = 6) and executive function (*n* = 5).

The studies included in this review examined three cognitive domains (Figure 4): (1) spatial memory [55–60], which is the knowledge of spatial orientation in an environment; (2) working memory, processing speed, and learning [41, 53, 57, 61–66], which involves manipulating multiple pieces of information in order to perform a task; and (3) executive function [41, 53, 63, 67, 68], which involves complex cognitive processes such as planning and problem solving.

#### 3.2.1. Spatial Memory

The effects of AE on spatial memory have only been tested in animals and five of the six studies reported improvements following AE (Figure 4(a)). Of these five, only one employed VE [55] and reported significant improvements in spatial memory following 42 days of VE in mice, beginning 7 days after a focal lesion. With respect to FE protocols (Table 2), low-to-moderate intensity exercise for two to four weeks, 2–4 days following a focal lesion, significantly improved spatial memory compared to sedentary controls [56, 57, 60]. However, progressive moderate-to-high intensity FE did not improve spatial learning after 4 weeks [57] but did so after twelve weeks (3x/week) of training [58], following focal or global ischemia, respectively. Furthermore, four of these studies tested spatial memory using the Morris Water Maze [55, 56, 58, 60], which controls for the confounding effect that improved locomotor ability could have on performance of overground spatial memory tests.

Consistent findings in animal models suggest that very intense AE protocols 2–4 days after stroke may not benefit spatial memory. For example, four weeks of forced swimming (20 min/day, 5 days/week, 24 hours after stroke) [59] did not improve spatial memory in the Morris Water Maze test. In addition, Shih and colleagues [60] found that low intensity, beginning 24 hours after stroke, but not high intensity swimming improved spatial memory. Similarly, four weeks of high intensity treadmill training beginning four days after ischemia failed to show improvements in spatial memory [57]. Given the association between excessive stress and spatial memory [74], it may be that protocols employing high intensity interventions, such as forced swimming, could be too stressful a manipulation for the animals. In summary, synthesized findings suggest that FE (short and longer term) and VE of moderate intensities improve spatial memory after stroke in animals (Table 2). It is important to note that since no follow-up assessments were completed after the intervention ceased, the durability of the benefits is not known.

#### 3.2.2. Working Memory, Processing Speed, and Learning

Nine studies, including 6 clinical trials, investigated the effects of AE on tests of memory, learning, and/or processing speed (Figure 4). In rodents with global ischemia, short term (10 days) low intensity FE significantly improved memory as assessed by the step-down inhibitory task [61]. Longer term interventions of four weeks, of both low [57, 62] and high intensity exercise, also significantly improved memory after global [62] and focal [57] ischemia when assessed by the same test [62], the object recognition test and the passive avoidance test [57]. These findings suggest that AE improved learning and memory in animals, regardless of the intensity or duration at least when initiated soon after stroke (Table 2).

Clinical trials of adults with chronic stroke showed inconsistent results; however we observed some patterns emerging (Figure 4(b)). In terms of AE intensity, less rigorous AE, either a single 20-minute bout of moderate intensity BWSTT [53] or a six-month, twice-per-week, low intensity program [63], did not significantly improve working memory when tested by the symbol digit test [53] or the digit span backward test [63]. However, following light-to-moderate intensity recumbent stepping for thirty minutes, thrice weekly for twelve weeks, Kluding and colleagues [64] reported a significant increase in working memory compared to controls as measured by the backward digit span test.

With respect to the effects of AE on learning, the benefits of AE appear to be short term. After eight weeks of moderate, thrice weekly recumbent cycling, Quaney and colleagues [41] reported significant improvements in motor learning as demonstrated by two nonparetic arm tasks: the serial reaction time task and the predictive grip force modulation test, a skilled task that assesses conditional learning ability of the relationship between object color and weight. However, they found no significant differences compared to controls when reassessed eight weeks after the end of the exercise protocol. Similarly, Rand and colleagues [63] reported significant improvements on the Rey Auditory Verbal Learning Test three months into their six months of mixed exercise protocol, but not at six months when the intervention ended.

Two clinical studies [65, 66] implementing AE for longer duration in combination with other therapies in chronic stroke reported more consistent benefits. In six participants with hemorrhagic stroke, a 12-week home program consisting of a combination of 30 minutes of AE (walking, treadmill, or stationary cycling) and 90 minutes of cognitive exercises per day exhibited a trend towards improved performance on the Mini-Mental Status Exam (MMSE) and Neurobehavioral Cognitive Status Examination, but not the Lowenstein Occupational Therapist Cognitive Assessment, when compared to those receiving traditional physiotherapy [66]. The other study implemented 25 extra intensive rehabilitation sessions over the course of one year after stroke, which included participant-directed AE, strength, balance, and postural control exercises in thirty-three stroke survivors with moderate-to-severe disability. The intervention significantly improved memory, language, visuospatial, and visual inattention measures (Alzheimer's Disease Assessment, Wechsler's Memory Scale, line bisection test, and Boston Naming Test), when compared to baseline within the group. Furthermore, memory scores were significantly higher compared to those who received traditional therapy [65]. It is important to note that neither of these studies included a control group and the effects of AE on long term outcomes once the intervention ceased are not known.

In summary, while a single bout of exercise or a low intensity exercise program did not influence working memory, moderate intensity and combination protocols reported improvements in multiple tests of memory and learning (Table 2). Future studies should examine AE (or AE in combination with other interventions) in people with subacute (rather than chronic) stroke with longer term follow-up.

#### 3.2.3. Executive Function

Executive function involves the control and management of various cognitive subprocesses to perform a complex task [75]. Five clinical studies examined the effects of AE on measures of executive function with equivocal results (Figure 4(b)) [41, 53, 63, 67, 68]. A single bout of moderate intensity BWSTT in adults with chronic stroke did not improve performance in Trail Making Test A or B, which measures aspects of executive function such as visual search speed and mental flexibility, nor does it significantly improve performance on the Paced Auditory Serial Addition Test, a measure of attention [53]. In a longer exercise intervention of eight weeks of moderate intensity, thrice weekly stationary cycling, Quaney and colleagues [41] found no significant differences in the Wisconsin Card Sorting Test, a measure of cognitive flexibility, the Stroop test, a measure of cognitive flexibility, processing speed, and attention, or the Trail Making Test when compared to controls.

Three studies reported significant beneficial effects of exercise on executive function. Rand and colleagues' [63] six-month mixed exercise intervention resulted in a short term significant improvement (after three months) in the walking while talking test—a measure of divided attention. However, these results were not significantly different from baseline when measured again at six months. Although it was noted that a small sample size (*n* = 11) may have limited the statistical power, no significant improvements occurred in the Trail Making Test but small improvements were reported in the Stroop test. A shorter, eight-week, AE intervention also resulted in significant improvement in overall Addenbrooke's Cognitive Examination-Revised scores, compared to physiotherapy alone [68].

Perhaps of the most interest are the results of a pretest/posttest study performed by Marzolini and colleagues [67], which involved the most robust AE paradigm of the human-based studies in this review. A group of 41 adults with stroke of varying durations (10–650 weeks after stroke) performed low-to-moderate intensity AE five times a week and resistance training three times a week for six months. AE included treadmill or overground walking and recumbent or upright cycling with sessions that progressed from 20 to 60 minutes in duration. At the end of the intervention, significant improvements occurred in the visuospatial/executive and attention/concentration scales of the Montreal Cognitive Assessment. Furthermore, overall scores on the Montreal Cognitive Assessment were also significantly improved, and the percentage of patients classified by the measure as having mild cognitive impairment significantly decreased from 65.9% to 36.6%. However, the role of resistance training in conjunction with AE in this intervention is uncertain.

In summary, both short and long term AE positively influenced memory and learning in animals and, to some extent, in humans. There was little evidence that AE alone improved executive function in people with stroke, but combination therapies and more intensive, longer duration (>3 months) exercise programs [67] were very promising (Table 2). Since rehabilitation is concerned with long term recovery, future studies should demonstrate that the benefits of AE extend beyond the end of the intervention.

## 4. Discussion

Previous reviews of AE concluded that early-initiated moderate intensity exercise enhanced neurotrophin expression, stimulated synaptogenesis and dendritic branching, and protected perilesional tissue against inflammation and oxidative injury mainly in animal models [69, 70]. Through these mechanisms, we proposed that AE could have beneficial effects on outcomes other than locomotion and physical fitness. We wished to uncover potentially important findings in animal studies and early clinical studies in order to inform future investigations of the effects of poststroke AE on cognitive and complex motor recovery. Our search resulted in 17 human trials with the vast majority of research being undertaken in animal models (*n* = 35). Furthermore, although all animal studies employed exercise within days after stroke induction, we found only a few clinical studies examining AE during the postacute, rehabilitative phase (<6 months) after stroke. This disparity made it difficult to compare findings. Future research should bridge this temporal gap by examining the effects of AE beginning at earlier time points after clinical stroke, since there is evidence for the benefits of early rehabilitation [39, 46, 68]. Furthermore, most studies confirmed that AE improved lower extremity function with fewer demonstrating that AE could influence recovery of more complex movement.

**Figure 5:**
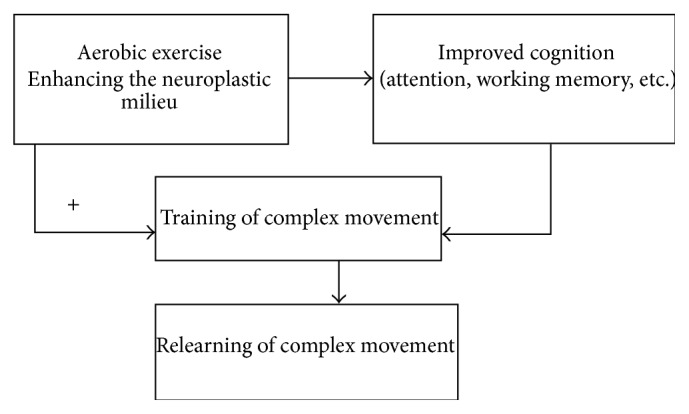
Proposed model of how aerobic exercise affects stroke outcomes. Aerobic exercise, by enhancing the neuroplastic milieu (increased neurotrophins, synaptogenesis, and dendritic branching and reducing oxidative stress), may improve cognition. AE, when combined with skilled task training, may potentiate relearning and recovery in two ways, (1) indirectly by improving attention and working memory to enhance relearning or (2) directly by enhancing activity-induced plasticity.

Although it is not clear exactly how AE could enhance outcomes unrelated to locomotion or cardiovascular fitness, based on our findings it appears that AE could be enhancing the neuroplastic milieu within the brain after stroke by making the brain more amenable to modification and new learning. Alternatively, AE may be enhancing the attention, concentration, and learning mechanisms that underlie relearning of complex skill. Researchers have shown that, at least in uninjured brains, AE enhances corticospinal excitability [76] and long term potentiation-like plasticity [77].  Figure 5 outlines these proposed mechanisms.

### 4.1. Complex Upper Extremity Movement Was Relatively Resistant to AE Effects

AE did not seem to improve upper limb function; however, there were promising results in animal models for moderate intensity combined approaches involving exercise plus skill training [29]. Gross sensorimotor function showed more consistent benefits in animal models which could be expected since forelimb movement is required for quadruped ambulation used in the AE protocols. There is more research required to determine if AE influences relearning of fine digit control. It is also important to note that there have been no studies investigating the effects of AE on fine motor control or relearning of lost upper extremity movement using longer duration protocols (>5 weeks in animals, >4 weeks in clinical trials).

### 4.2. AE Improves Memory and Learning

Our review supported the positive influence of poststroke AE on cognitive performance, although the effect was not universal. In general, moderate intensity AE programs appeared beneficial in cognitive tests in animals but less so in more complex executive tasks in clinical studies. In animals, both short term FE and longer term FE and VE of various intensities improved spatial memory test performance [55–58, 60]. However, when AE paradigms were too stressful, it adversely affected specific cognitive outcomes like spatial memory [57, 60]. In contrast, learning and working memory in animals were improved regardless of the intensity or the duration of the intervention [57, 61, 62]. Compared to animal models, clinical trials reported that combined interventions were most promising for cognitive improvements even if they were more intense, frequent, and longer in duration (>3 months) [65–67]. The biggest challenge with combined interventions is that it is difficult to decipher to what extent each ingredient of the protocol accounted for the observed benefits. Nonetheless, there is emerging evidence that combining AE with functional exercises, resistance training, or even cognitive training could achieve better cognitive rehabilitation outcomes than AE training alone.

### 4.3. AE Consistently Improves Lower Limb Recovery

This review confirmed that primarily low-to-moderate FE (or VE) improved lower extremity coordination in rodents with stroke, which is not surprising considering that most training involves overground locomotion and therefore would likely improve performance in the same task. The timing of initiating the intervention affected functional outcomes as well, with early onset training (<5 days) appearing to be most beneficial [17–19, 21–24, 31, 32, 37, 38]. Investigators employed a variety of tests to measure lower extremity and balance recovery in animal models (Figure 2(a)), some more challenging (and likely more sensitive) than others. For example, the rotarod was used to assess coordination and balance on a moving surface while the horizontal bar assessed the same on a static surface [78]. The choice of outcome measure may impact the ability to detect subtle changes due to the intervention.

In contrast to the animal models, clinical trials almost exclusively reported positive outcomes in complex lower extremity mobility and balance following poststroke AE. Interestingly, the frequency (3–5 times a week), intensity (low, moderate, or high), duration (4 weeks–3 months), and timing of intervention initiation (1 to >12 months after stroke) did not appear to have an effect on the outcomes. However, studies that implemented a control group, that received conventional therapy instead of AE [40], or that received a home-based exercise protocol [47] reported positive outcomes for complex lower extremity function and balance as well, albeit to a lesser degree. It is worth noting that, following AE, only one trial reported sustained improvement in balance (8 weeks after intervention) [41]. While another study reported improvements in both locomotor coordination and balance approximately 1 month after intervention, both the AE and the strength and balance exercise groups improved compared to conventional therapy. The effects of AE specifically could not be determined [47]. Follow-up assessment is a critical study design component in order to assess the sustained effects of AE-based rehabilitation since rehabilitation aims to promote long term recovery, not only short term improvements in performance.

## 5. Conclusion and Future Directions

In this review, we highlighted some key findings from studies assessing the effects of AE on complex physical and cognitive outcomes. Strongest evidence exists for lower extremity and balance outcomes which have some bias since the intervention and the outcome are related tasks. In order to confirm that AE could potentially “prime” the brain and influence neuroplasticity [12], future studies need to demonstrate that AE improves tasks that are unrelated to locomotion such as fine digit control or executive function. More than 1 in 3 patients suffer from stroke-related cognitive impairments that can significantly affect motor relearning after stroke, resulting in increased disability and institutionalization [79, 80]. As a result, identifying how the timing of starting an AE-based rehabilitation affects cognitive outcomes after stroke will be critical in developing appropriate intervention strategies. Animal models offer many useful insights that can be translated into clinical trials, but the temporal gap of when AE was initiated is very different between the two (days after stroke in animal models versus months to years in trials). Clinical studies must be undertaken to examine early AE intervention when the brain may be most amenable to change.

## Figures and Tables

**Table 1:** Summary of search results by theme (clinical trials in bold lettering).

Motor performance	Balance and lower limb coordination	(Sakakima et al., 2012) [17]; (Ma et al., 2013) [18]; (Matsuda et al., 2011) [19]; (Park et al., 2010) [20]; (Chang et al., 2009) [21]; (Chang et al., 2011) [22]; (Mizutani et al., 2010) [23]; (Yang et al., 2012) [24]; (Risedal et al., 2002) [25]; (Dahlqvist et al., 2003) [26]; (Johansson and Ohlsson, 1996) [27]; (Ding et al., 2004) [28]; (Ploughman et al., 2007) [29]; (Buiatti de Araujo et al., 2008) [30]; (Zhang et al., 2013) [31]; (Zhang et al., 2013) [32]; (Nielsen et al., 2013) [33]; (Mizutani et al., 2013) [34]; (Mizutani et al., 2014) [35]; (Mizutani et al., 2015) [36]; (Chang et al., 2014) [37]; (Ikeda et al., 2013) [38]; **(Straube et al., 2014) [39]; (Jin et al., 2013) [40]; (Quaney et al., 2009) [41]; (Gama et al., ** **2015)** ** [42]; (Chen et al., 2014) [43]**; **(Batcho et al., 2013) [44]; (Yang et al., 2014) [45]; (Yang et al., 2010) [46]; (Nadeau et al., 2013) [47]**
	Upper limb movement	(Ploughman et al., 2007) [29]; (Maldonado et al., 2008) [48]; (Park et al., 2010) [20]; (G. Kim and E. Kim, 2013) [49, 50]; (Heo and Kim, 2013) [51]; (G. Kim and E. Kim, 2013) [49, 50]; (Schneider et al., 2014) [52]; **(Ploughman et al., 2008) [53]**; **(Rabadi et al., 2008) [54]**

Cognitive performance	Spatial memory	(Luo et al., 2007) [55]; (Zhang et al., 2012) [56]; (Shimada et al., 2013) [57]; (Cechetti et al., 2012) [58]; (Song et al., 2012) [59]; (Shih et al., 2013) [60];
	Working memory, processing speed, and learning	(Sim et al., 2004) [61]; (Sim et al., 2005) [62]; (Shimada et al., 2013) [57]; **(Ploughman et al., 2008) [53]; (Quaney et al., 2009) [41]**; **(Rand et al., 2010) [63]; (Kluding et al., 2011) [64]; ** **(Pyäriä** ** et al., 2007) [65]; (Pyun et al., 2009) [66]**
	Executive function	**(Ploughman et al., 2008) [53]; (Quaney et al., 2009) [41]; (Rand et al., 2010) [63]; (Marzolini et al., ** **2013)** ** [67]; (El-Tamawy et al., 2014) [68]**

**Table 2:** Synthesized findings of optimal parameters to affect stroke outcomes.

Parameter	Outcome					
	Balance/lower extremity	Upper limb Gross motor	Upper limb Fine control	Spatial memory (animals only)	Working memory, processing, learning	Executive function (clinical only)
Frequency	*Animals* 5–7x/week *Clinical* 3x/week	*Animals* 5–7x/week *Clinical* Unknown	Unknown	5–7x/week	*Animals* Daily *Clinical* 3x/week	3–5x/week

Intensity	*Animals/clinical* Moderate-high	*Animals* Moderate-high *Clinical* Unknown	Unknown	Low-moderate	*Animals* Low-moderate *Clinical* Moderate-high	Moderate-high

Time/duration	*Animals* 20–30 min 2–4 weeks *Clinical* 60 min 4–8 weeks	*Animals* 20–30 min Duration unknown *Clinical* Unknown	Unknown	20–30 min 2–4 weeks	*Animals* 30 min 10 d or 4 weeks *Clinical* 20–60 min 8 weeks	30–60 min 8 weeks or 6 months

Type/modality	*Animals* FE and VE *Clinical* Mixed	*Animals* FE *Clinical* Unknown	Unknown	FE and VE	*Animals* FE *Clinical* Mixed	Mixed

Initiation after stroke	*Animals* 1–5 days *Clinical* >4 months	*Animals* 1–5 days *Clinical* Unknown	Unknown	2–4 days	*Animals* 2–4 days *Clinical* >4 months	>3 months
